# Tele-Exercise as a Promising Tool to Promote Exercise in Children With Cystic Fibrosis

**DOI:** 10.3389/fpubh.2018.00269

**Published:** 2018-09-28

**Authors:** Jen Jen Chen, Dan M. Cooper, Fadia Haddad, Anna Sladkey, Eliezer Nussbaum, Shlomit Radom-Aizik

**Affiliations:** ^1^Department of Pediatric, Pediatric Exercise and Genomics Research Center, School of Medicine, University of California, Irvine, Irvine, CA, United States; ^2^Miller Childrens' and Womens' Hospital, Long Beach, CA, United States

**Keywords:** exercise training, fitness, exercise medicine, pediatrics, physical activity

## Abstract

**Introduction:** Cross-infection risk from contact exposure limits exercise opportunities in children with cystic fibrosis (CF). The purpose of this study is to evaluate the feasibility of a new live-streamed platform which delivered supervised and interactive group exercise sessions to CF children via digital devices while avoiding contact exposure.

**Methods:** Ten CF children participated in a 6-week tele-exercise program. The program consisted of three 30-min sessions per week for a total of 18 sessions and included aerobic, resistance, and flexibility exercises. Sessions were streamed via a HIPAA compliant VSee telemedicine platform. Instructors and participants were able to interact in real-time online. Heart rate (HR) monitors were used to evaluate exercise intensity with a goal of moderate-vigorous physical activity ≥10 min, 70% of the sessions. System usability scale (SUS) and qualitative questionnaires were used to gauge participants' satisfaction and feedback.

**Results:** On average participants attended 85% of the sessions. For the overall sessions participants exercise 21.1 ± 6.9 min at moderate-vigorous physical activity. Nine out of 10 participants used the exercise platform without parental guidance. Qualitative questionnaire and System Usability Scale (SUS) indicated that all participants enjoyed the tele-exercise program and highly rated the exercise platform 90.8 out of 100 (passing > 68).

**Conclusions:** Tele-exercise platform is a promising new approach to promote exercise in children with CF. The online platform allows supervised virtual group exercise experience with optimal participation and no risk for cross-infection. This approach might prove to be useful in enhancing the use of exercise as therapy in children with CF.

## Introduction

Fitness in patients with cystic fibrosis (CF) is associated with increased survivability (1) but only 60% of CF centers follow the recommendation for annual fitness tests (2). Promoting exercise at home has yet to be adopted as an integral component of CF care. Summer residence exercise programs for children with CF, in which the exercise regimens and social milieu can be geared to the needs of these patients with CF, lead to significant improvement in exercise parameters and well-being (3). However, concerns regarding the cross-infection risk have almost completely eliminated such group activities preventing CF patients from receiving the benefit of peer interactions which can promote physical activity in youth (4).

There is an emerging body of literature suggesting that telemedicine has been proved useful in providing home rehabilitation for chronic diseases (5, 6). Tele-exercise has the promise to provide a supervised group exercise experience while mitigating the potential problem of cross-infectivity. No studies have been done to assess the feasibility of tele-exercise in CF. Our pilot study was the first to examine the implementation of an interactive tele-exercise program with real-time wearable device data collection in pediatric CF patients. Our goal was to evaluate the feasibility of a streaming software program and remote monitoring as a convenient and accessible method for pediatric CF patients in order to maintain desired level of physical activity.

## Materials and methods

### Participants

**Figure 1:**
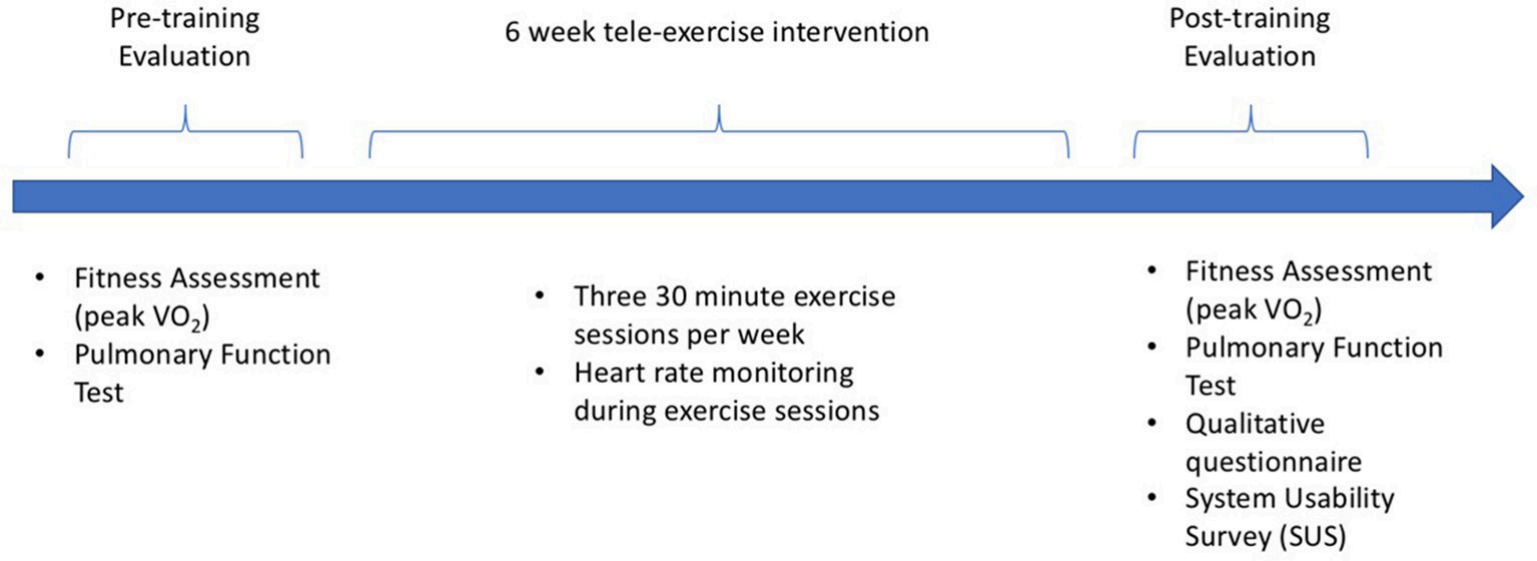
Overview of study design.

Ten children and adolescents with CF 8–20 years of age participated in this study. Table 1 illustrates the anthropometric, physiological and genetic characteristics of the participants. Those who had severe pulmonary disease (FEV1 < 40%), recent pulmonary exacerbation within the last month, or unable to perform exercise as determined by their physician were excluded from the study. Figure 1 depicts an overview of study design. The study was approved by the Institutional Review Board at Memorial Care Health System, and written informed assent and consent was obtained from all participants and their parents upon enrollment.

**Table 1:** Participants anthropometric, physiological, and genetic characteristics.

**Subject ID**	**Age range**	**Genetics**	**Baseline BMI % ile**	**Baseline Peak VO_2_ (mL/kg/min) (%predicted)**	**Post-training peak VO_2_ (mL/kg/min) (%predicted)**	**Baseline FEV1 (L)**	**Post-training FEV1**	**Sessions attended(Total = 18)**
1	11–15	F508, p.R1066H, P.L1324P	63	27.4 (76)	28.8 (79)	86	88	17
2	11–15	F508, F508	33	33.9 (80)	35.2 (89)	94	98	5
3	16–20	C.2988 + IG > A, N1303K	74	28.4 (83)	27.3 (77)	91	83	17
4	11–15	F508, 1717-IG->A	17	43.8 (97)	36.5 (83)	74	76	14
5	6–10	F508, C948delT (1078delT)	61	40.1 (102)	41.7 (99)	88	85	16
6	16–20	3,120 + G7A/3876delA	76	23.9 (70)	25.7 (75)	65	52	13
7	16–20	F508, F508	17	28.7 (81)	29.2 (83)	75	72	17
8	11–15	F508, F508	81	32.3 (88)	33.6 (92)	110	110	14
9	11–15	W1282x/2183 delAA>G	72	44.1 (98)	46.2 (103)	86	88	15
10	16–20	F508, F508	17	27.9 (71)	23.1 (58)	81	63	18

### Anthropomorphic assessment

Standard calibrated scales and stadiometers were used to determine height and body mass. Body mass index [BMI = wt/ht^2^ (kg/m^2^)] percentile was calculated using the published standards from the Centers for Disease Control, National Center for Health Statistics (7).

### Fitness assessment

Each participant performed a ramp-type progressive cycle ergometer cardiopulmonary exercise test (CPET) using the Medgraphics Ultima™ CardiO2® gas exchange analysis system before and after the 6-week tele-exercise program. Following 1 min of unloaded pedaling, the work rate (WR) was increased at 10–15 watts/min increments to the limit of the subject's tolerance. Gas exchange was measured breath-by-breath and peak VO_2_ was calculated using standard methods (8, 9). There is currently no validated, universally-accepted respiratory exchange ratio (RER) cutoff in children for the determination of peak VO_2_. We used RER > 1.0, a criterion used by Rowland and coworkers (10). Percent predicted peak VO_2_ was calculated based on Medgraphics pediatric norms. Pulmonary function testing was done prior to the fitness test to measure maximal expiratory flow rates (FEV1).

### Software setup

Each participant was instructed how to download and setup VSee, a HIPAA compliant and encrypted telemedicine program. A member of the research team was available to visit the participant's homes to help set up or for troubleshooting. Before starting the exercise program, a test run was performed on VSee with research staff and the participant to ensure that lighting and internet connection was optimal.

### Exercise program

The exercise program consisted of three 30-min session per week over 6 weeks for a total of 18 sessions. One participant wanted to volunteer but, for personal reasons, did not want to exercise with other children even virtually. The remaining nine participants were divided into three groups. Each session was streamed in real-time using VSee and allowed live interaction among the instructors and participants. Sessions consisted of 3–4 min of warmup, 20–25 min of aerobic and plyometric exercises, ending with 3–4 min of stretching and huff coughing.

Exercise intensity during the sessions was assessed using Polar heart rate (HR) monitors. Moderate physical activity (PA) was defined as 60–75% of estimated maximum HR of 200 bpm. Vigorous PA was defined as >75% of estimated maximum HR. The number of minutes participants exercised at moderate-intensity (120–150 bpm), vigorous intensity (150–180 bpm) and >180 bpm was recorded. The aim was to have at least 10 min out of the 30-mi exercise session at moderate-vigorous exercise intensity in at least 70% of the exercise sessions. Data is presented as means ± SD.

### Questionnaires

Following the tele-exercise program, participants completed a system usability scale (SUS) to evaluate the user's satisfaction and the technological effectiveness of the software. SUS scores range from 0 to 100 with a passing score > 68 (11). The SUS is a widely used assessment tool to rate the perceived usability of computing products such as telemedicine platforms (12). Participants also answered a qualitative questionnaire regarding their thoughts about the program:

What was your most/least favorite part of the program and why?
What were the most/least favorite exercises and why?
Would you participate in a similar program again?
What type of changes have you seen because of the program, if any?
How has the program helped you?

## Results

Participants attended on average 15 (85%) of the sessions with nine participants attending 13 (75%) or more of the 18 exercise sessions (Table 1). One participant attended 5 (28%) of the sessions due to scheduling conflicts with school and extracurricular activities. Each session lasted about 30 min. 9 out of the 10 participants achieved the target HR goal and participants overall spent 21.1 ± 6.9 min on moderate-vigorous PA. Participants exercised 15.3 ± 3.4 min at moderate intensity (120–150 bpm), 5.8 ± 4.6 min at vigorous intensity (150–180 bpm) and achieved maximum HR of 159.5 ± 10.4 bpm. Baseline peak VO_2_ was on average 84.6 ± 11.3% predicted and did not change following the 6-week exercise intervention. No significant differences beyond normal short-term variability were observed between pre- and post-exercise program in pulmonary function test (PFT) (13). The participant who had the largest relative reduction in FEV1 and peak VO_2_ at post-training also had respiratory illnesses on the day of the test (Table 1).

Using the System Usability Scale, the exercise program on the VSee platform was highly rated by the participants with a score of 90.8, suggesting that the software platform and exercise program was easy to use without requiring outside guidance or intervention. Qualitative questionnaire indicated that all participants liked the tele-exercise program and wanted to continue with the exercise sessions. Participants enjoyed having the motivation to exercise, the opportunity to be led by an instructor, and most of them cited the social benefits of a group setting as their favorite parts of the program:

“Love chatting with everyone.”

“Talking to people because it made the sessions more fun.”

    “Seeing friends and the instructor.”

    “I love working out with everyone even though I'm exhausted, I have fun, it makes me not hate working out.”

    “I like socializing with everyone.”

    “All of [it] because it is exercise [I] would not have otherwise gotten.”

Only one participant had a technological critique because her “screen kept freezing.” Participants provided statements on how the program had helped them:

“Helped me exercise instead of watching TV.”

    “It made me ‘exercisable’ and got to see the doctor.”

    “It helped me because I'm always lazy to work out or do it myself.

    “Strengthened muscle around my knees so [they] had more stability, drastically decreasing my knee pain. Also noticed that the exercises got easier as the program went by.”

    “Great shape and it was fun.”

    “Less coughing, less lung issues [that] negatively impact [my] daily life…, more positive, better sleep.”

## Discussion

The aim of this study was to evaluate feasibility of an online interactive platform as a way to deliver and promote exercise in children with CF. Nine out of 10 participants attended most of the exercise sessions, suggesting that streaming tele-exercise sessions is a viable and convenient method to encourage physical activity without subjecting them to the cross-infection risks associated with in-person group activity. The tele-exercise platform allows for flexible scheduling and is accessible from home, bypassing transportation barriers. From most of the participants' observations, the appeal of the tele-exercise program seemed to be centered on the group supervised training.

Incorporating remote monitoring was essential for instructors to adjust the exercise prescription based on the participant's performance. During the exercise session, instructors would pause periodically to ask participants for their HR readings. In this study, nine participants achieved target HR goal, while one was just below (9.4 ± 5.3 bpm) the target HR goal. An improvement to the next iteration of the tele-exercise program would be the addition of another platform that displays an overview of all participants' HR in real-time.

In contrast to traditional tele-rehabilitation studies using phone calls or video recording interventions, our study relied on the participants ability to use the VSee streaming platform as well as their home webcams. We found that regardless of age, participants had little trouble with signing on or using the platform and in fact, were overall very satisfied with their experience with the software. One participant reported difficulties with periodically having their screen frozen, which was attributed to the internet and could be avoided by checking the home's internet bandwidth prior to implementing the program to ensure that the VSee program could run with adequate resolution.

People with CF often feel isolated as they cannot interact face to face with their peers due to the high risk of cross contamination (14). There is evidence to support the idea that accountability is an important factor in exercise program adherence. Participants were motivated to log in because they had friends who share the same chronic disease to exercise with.

This study highlights the need for evaluating tele-exercise programs for children with CF. To improve aerobic fitness, the current program should be modified to include exercises at a higher target HR for longer duration. A larger study is needed to evaluate participants' retention in a longer program and evaluate additional physiological and psychological variables, such as muscle strength, agility, balance and cognitive function in different tele-exercise programs.

## Summary

Tele-exercise program is an innovative and promising approach to promote physical activity in children with CF through live interaction of streamed exercise sessions. Most participants felt they were part of a virtual exercise group. This platform is user-friendly and can be easily deployed for pediatric CF patients to provide supervised training, monitoring HR response and shortness of breath, while mitigating cross-infection risk. This approach may prove to be useful in enhancing the use of exercise as therapy in children with CF.

## Author contributions

JC, DC, AS, and SR-A: conception and design of study. JC and AS: data collection. JC, DC, FH, AS and SR-A: data analysis and interpretation. JC, DC, AS, and SR-A: drafting the article. JC, DC, AS, EN, and SR-A: critical revision of the article. JC, DC, FH, AS, EN, and SR-A: final approval of the version to be published.

### Conflict of interest statement

The authors declare that the research was conducted in the absence of any commercial or financial relationships that could be construed as a potential conflict of interest. The reviewer EH and handling editor declared their shared affiliation.

## References

[1] Nixon PA Orenstein DM Kelsey SF Doershuk CF. The prognostic value of exercise testing in patients with cystic fibrosis. N Engl J Med. (1992) 327:1785–8. 10.1056/NEJM199212173272504 1435933

[2] Barker M Hebestreit A Gruber W Hebestreit H. Exercise testing and training in German CF centers. Pediatr Pulmonol. (2004) 37:351–5. 10.1002/ppul.10430 15022132

[3] Blau H Mussaffi-Georgy H Fink G Kaye C Szeinberg A Spizer SA. Effects of an intensive 4-week summer camp on cystic fibrosis: pulmonary. Chest (2002) 121:1117–22. 10.1378/chest.121.4.1117 11948041

[4] Salvy SJ Bowker JC Germeroth L Barkley J. Influence of peers and friends on overweight/obese youths' physical activity. Exerc Sport Sci Rev. (2012) 40:172–32. 10.1097/JES.0b013e31825af07b 22543686 PMC7187637

[5] Thirumalai M Rimmer JH Johnson G Wilroy J Young HJ Mehta T. TEAMS (Tele-Exercise and Multiple Sclerosis), a tailored telerehabilitation mHealth app: participant-centered development and usability study. JMIR Mhealth Uhealth (2018) 6:e10181. 10.2196/10181 29798832 PMC5992455

[6] Marquis N Larivée P Saey D Dubois MF Tousignant M. In-home pulmonary telerehabilitation for patients with chronic obstructive pulmonary disease: a pre-experimental study on effectiveness, satisfaction, and adherence. Telemed J E Health (2015) 21:870–9. 10.1089/tmj.2014.0198 26075928

[7] National Center for Health Statistics Clinical Growth Charts. Hyattsville, MD: National Center for Health Statistics (2000). Available online at: http://www.cdc.gov/nchs/about/major/nhanes/growthcharts/clinical_charts.htm

[8] Swisher A Hebestreit H Mejia-Downs A Lowman J Gruber W Nippins M Exercise and habitual physical activity for people with cystic fibrosis: expert consensus, evidence-based guide for advising patients. cardiopulm Phys Ther J. (2015) 26:85–98. 10.1097/CPT.0000000000000016

[9] Hebestreit H Arets HG Aurora P Boas S Cerny F Hulzebos EH. Statement on exercise testing in cystic fibrosis. Respiration (2015) 90:332–51. 10.1159/000439057 26352941

[10] Rowland T Hagenbuch S Pober D Garrison A. Exercise tolerance and thermoregulatory responses during cycling in boys and men. Med Sci Sports Exerc. (2008) 40:282–7. 10.1249/mss.0b013e31815a95a7 18202574

[11] Brooke J System usability scale–a quick and dirty usability scale. Usability Eval Ind. (1996) 189:4–7.

[12] Cox N Alison J Button B Wilson J Holland A. Assessing exercise capacity using telehealth: a feasibility study in adult with cystic fibrosis. Respir care (2013). 58:286–90. 10.4187/respcare.01922 22711058

[13] Pellegrino R Viegi G Brusasco RO Crapo F Burgos R casaburi R. Interpretive strategies for lung function tests. Series “ATS/ERS task force: standardisation of lung function testing.” Eur Respir J. (2005) 26:948–68. 10.1183/09031936.05.00035205 16264058

[14] Saiman L Siegel J. Infection control in cystic fibrosis. Clin Microbiol Rev. (2004) 17:57–71. 10.1128/CMR.17.1.57-71.2004 14726455 PMC321464

